# Supplementary material for: Sexual dimorphism in adult Little Stints (Calidris minuta) revealed by DNA sexing and discriminant analysis
Source: PeerJ. 2018 Aug 8;6:e5367. doi: 10.7717/peerj.5367 (PMC6087421; doi:10.7717/peerj.5367)
Supplement: Supplemental Information 1 [file peerj-06-5367-s002.docx]

**Sexual dimorphism in adult Little Stints (*Calidris* *minuta*) revealed by DNA sexing and discriminant analysis**

**Supplemental Information**

**Table S1** Discriminant functions with various combinations of measurements for Little Stints before (D_5_, D_6_, D_7_) and after (D_8_, D_9_, D_10_) primary moult. N_F_ – number of females, N_M_ – number of males, THL – total head length, T+T – tarsus-plus-toe, tarsus – tarsus length, wing – wing length, bill – bill length.

| Discriminant function | N_F_ | N_M_ | Cut-off value | Correctly  sexed [%] | Cross validation  [%] |
| --- | --- | --- | --- | --- | --- |
| **before primary moult:** |  |  |  |  |  |
| D_5_ = –51.904 + 0.456 (wing) + 0.178 (T+T) | 69 | 86 | 0.19 | 80.6 | 80.0 |
| D_6_ = –47.932 + 0.428 (wing) + 0.340 (bill) | 70 | 86 | 0.18 | 81.4 | 80.8 |
| D_7_ = –52.263 + 0.443 (wing) + 0.229 (THL) | 70 | 86 | 0.17 | 80.8 | 79.5 |
| **after primary moult:** |  |  |  |  |  |
| D_8_ = –53.636 + 0.510 (wing) + 0.113 (tarsus) | 78 | 77 | -0.01 | 83.9 | 83.9 |
| D_9_ = –54.619 + 0.506 (wing) + 0.094 (T+T) | 79 | 77 | -0.03 | 84.0 | 84.0 |
| D_10_ = –53.119 + 0.472 (wing) + 0.329 (bill) | 79 | 77 | -0.03 | 85.3 | 85.3 |


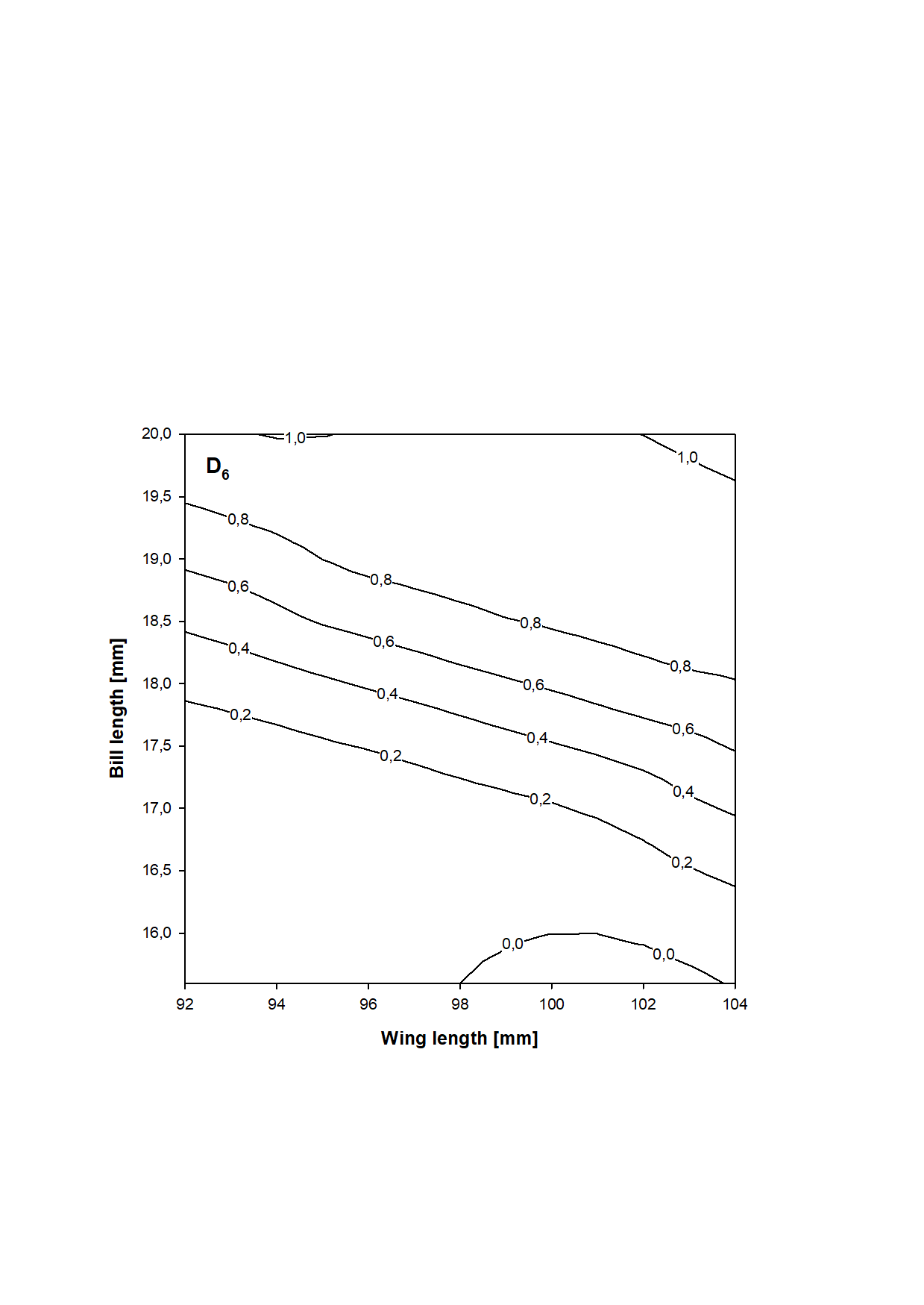


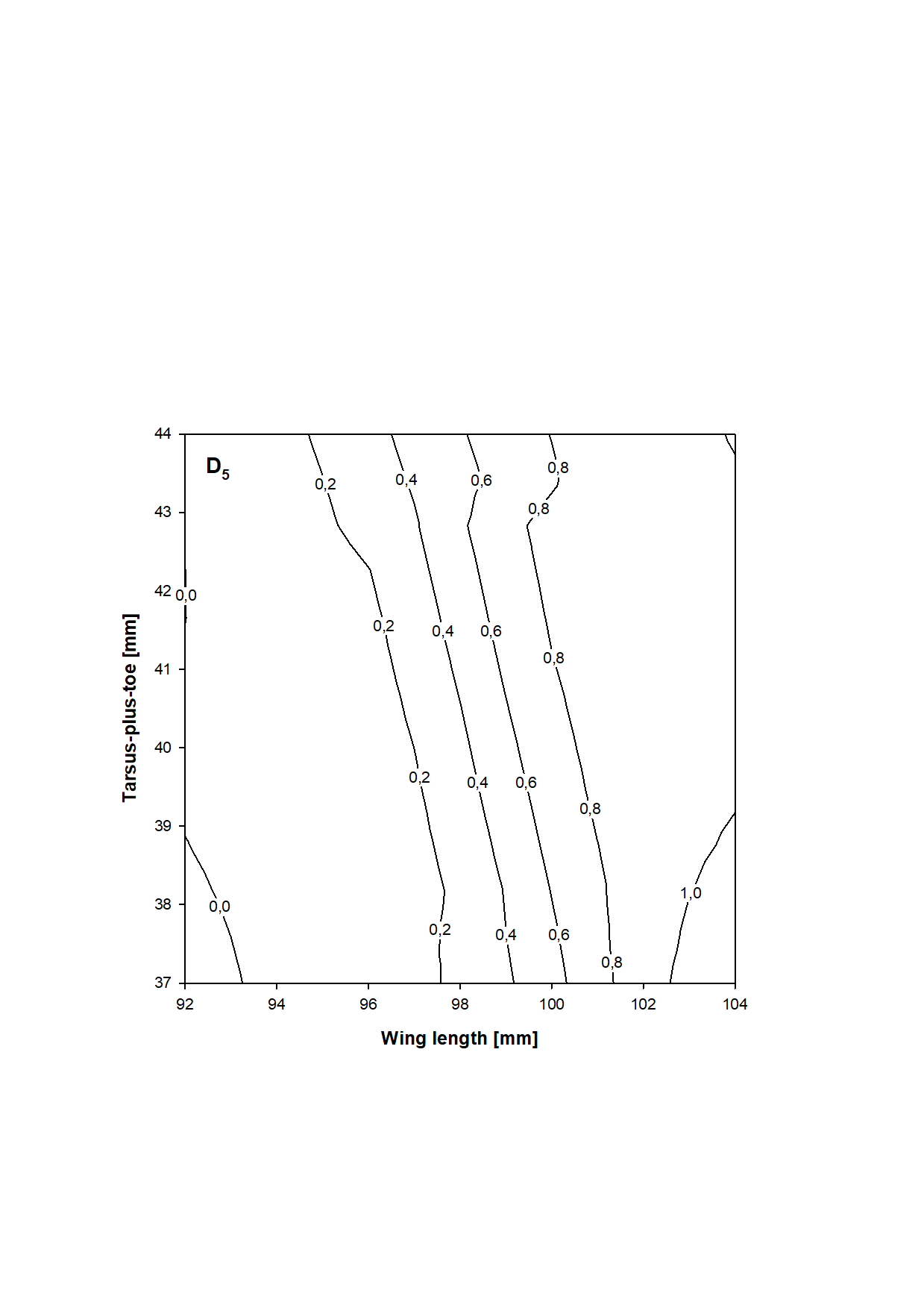

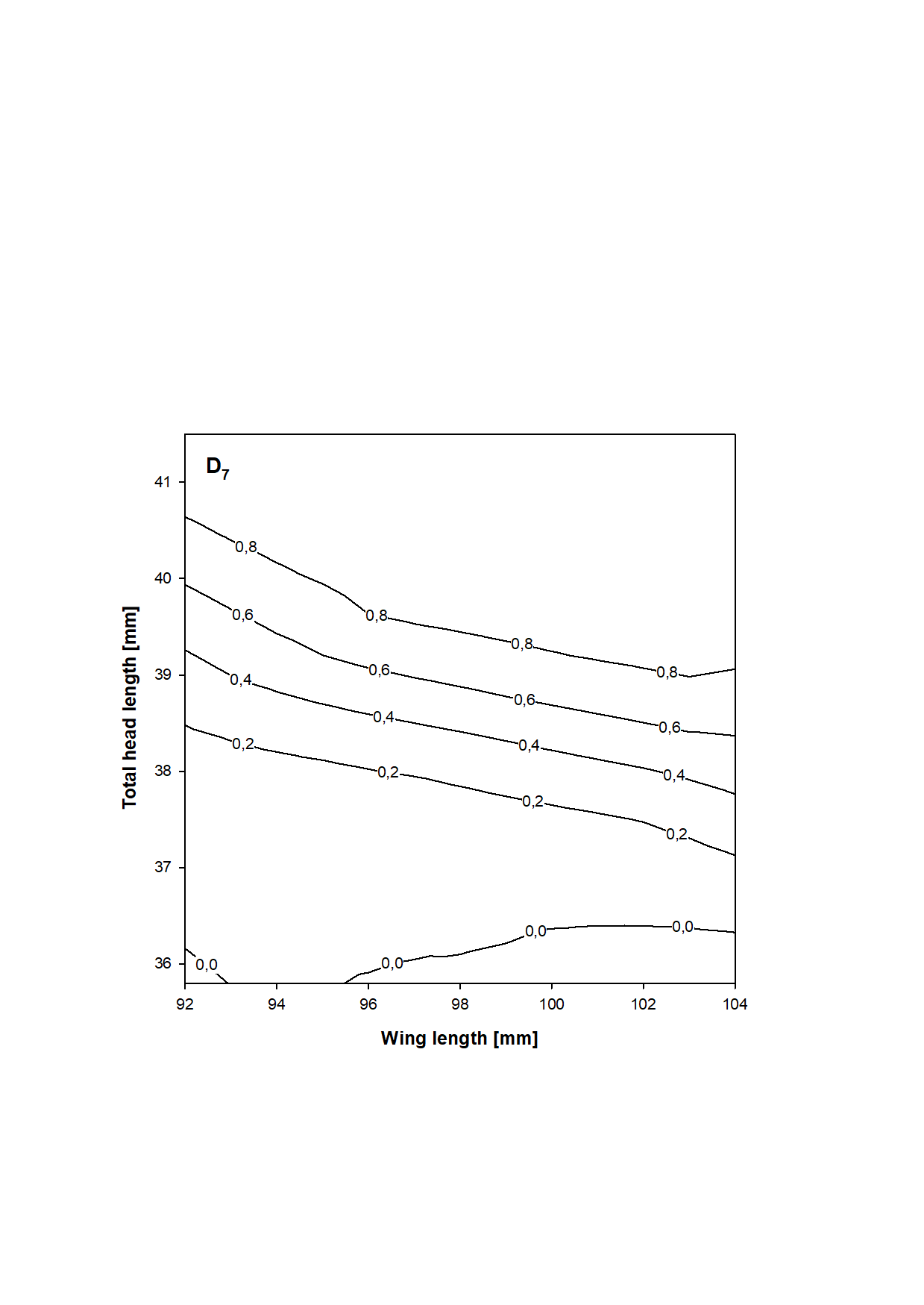


**Figure S1** Probability of being a female (lines and values show the upper limits) in relation to the combination of discriminating morphometric features according to additional discriminant functions (D_5_, D_6_, D_7_) for Little Stints before primary moult.


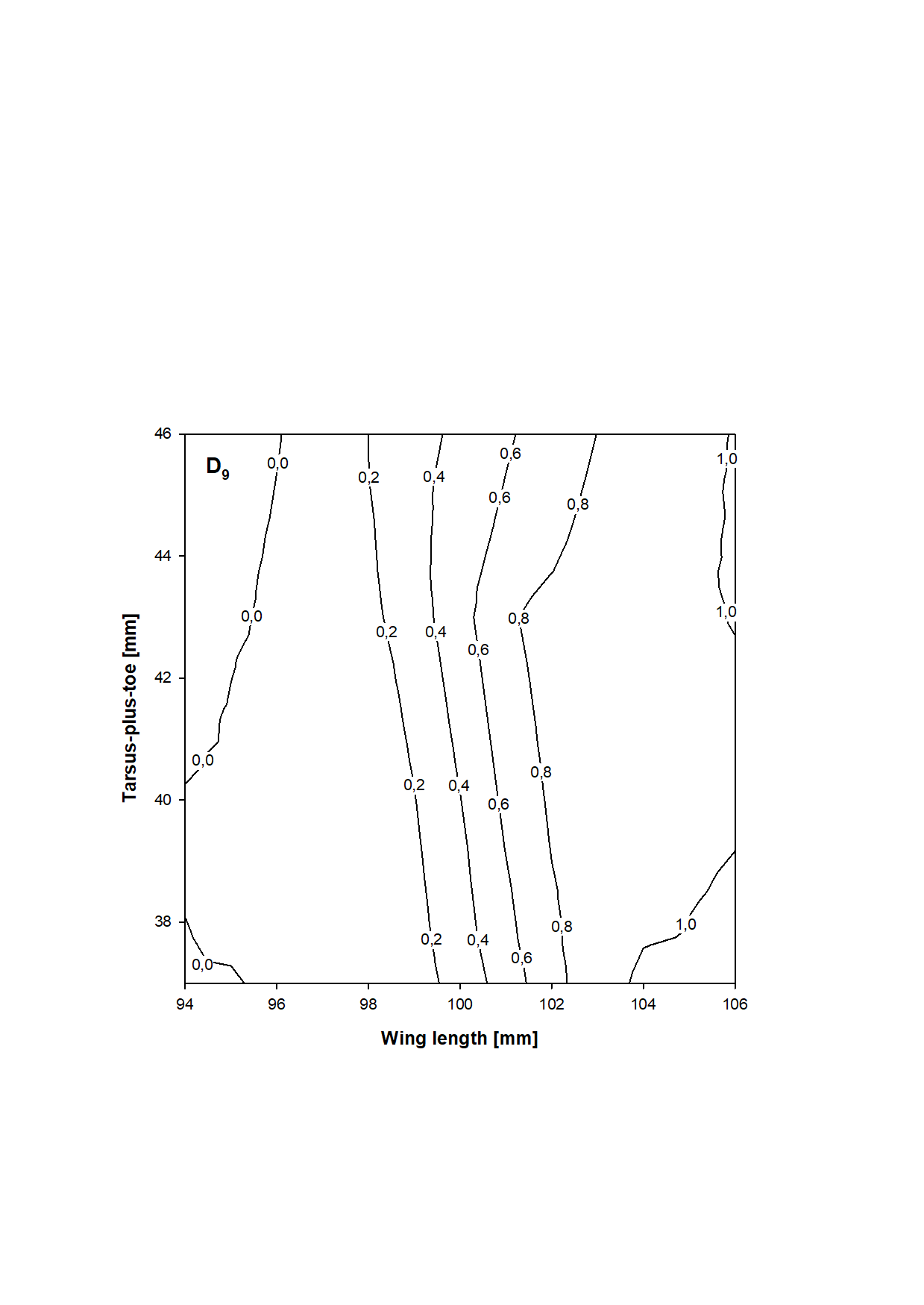


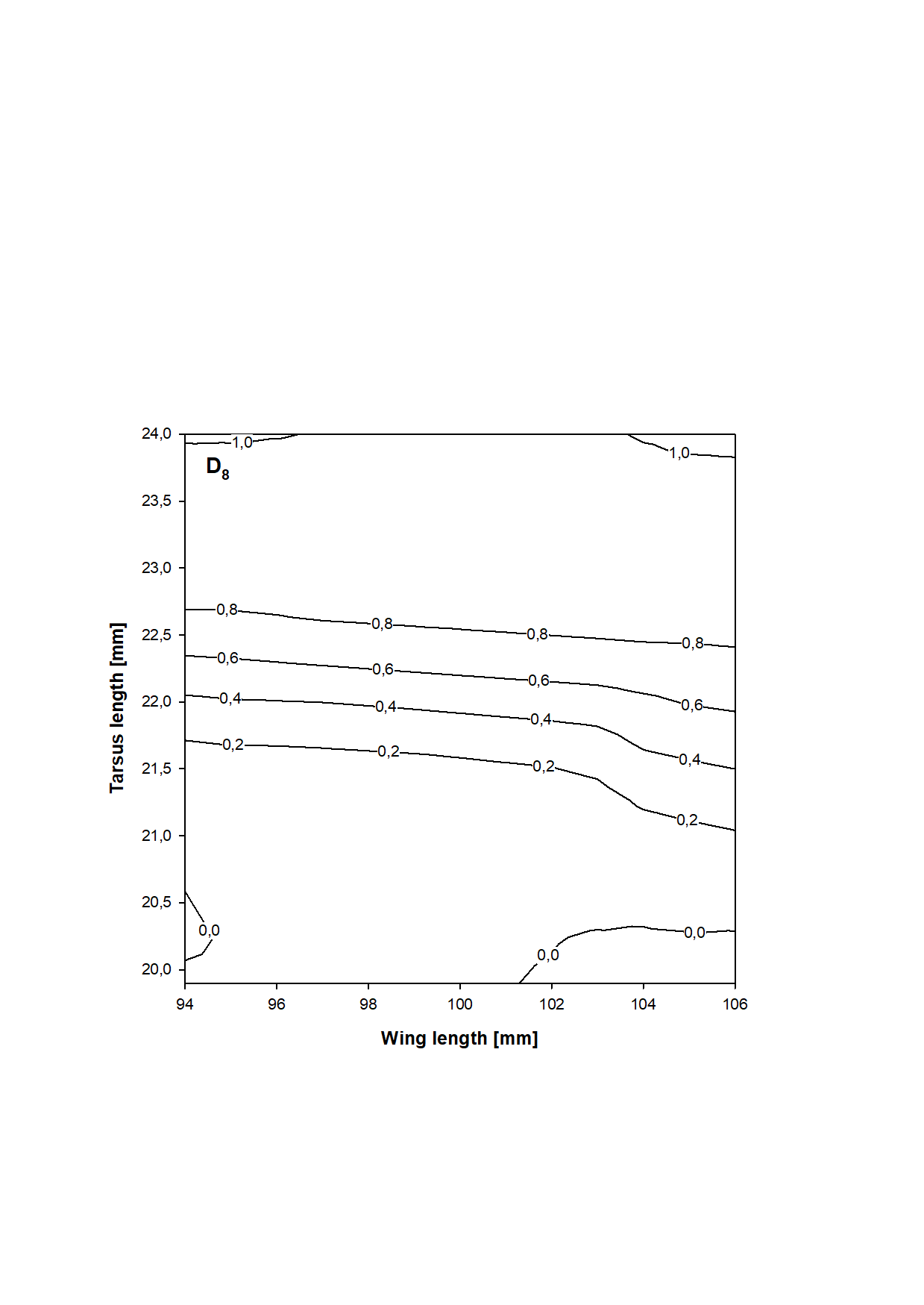

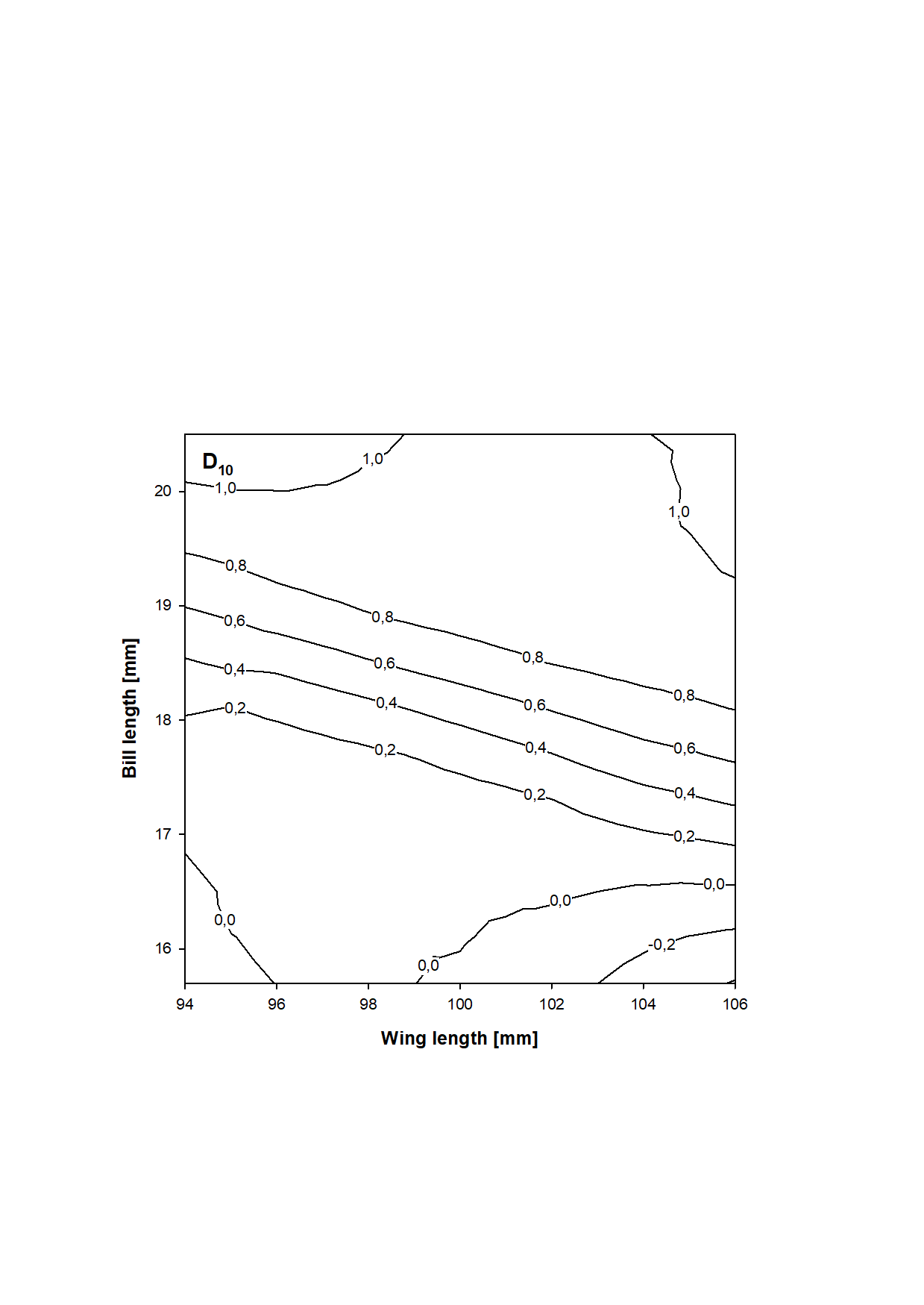


**Figure S2** Probability of being a female (lines and values show the upper limits) in relation to the combination of discriminating morphometric features according to additional discriminant functions (D_8_, D_9_, D_10_) for Little Stints after primary moult.
